# Supported local implementation of clinical guidelines in psychiatry: a two-year follow-up

**DOI:** 10.1186/1748-5908-5-4

**Published:** 2010-01-26

**Authors:** Tord Forsner, Anna Åberg Wistedt, Mats Brommels, Imre Janszky, Antonio Ponce de Leon, Yvonne Forsell

**Affiliations:** 1 Department of Public Health Sciences, Karolinska Institutet, Stockholm, SE-171 76, Sweden; 2 Department of Clinical Neuroscience, Section of Psychiatry St Göran's Hospital, Karolinska Institutet, Stockholm, SE-112 81, Sweden; 3 Medical Management Centre, Department of Learning, Informatics, Management, and Ethics, Karolinska Institutet, Stockholm, SE- 171 77, Sweden; 4 Department of Public Health, University of Helsinki, Helsinki, Finland; 5 Department of Public Health Sciences, Karolinska Institutet, Stockholm, SE-171 76, Sweden; 6 Department of Epidemiology, Rio de Janeiro State University, Brazil

## Abstract

**Background:**

The gap between evidence-based guidelines for clinical care and their use in medical settings is well recognized and widespread. Only a few implementation studies of psychiatric guidelines have been carried out, and there is a lack of studies on their long-term effects.

The aim of this study was to measure compliance to clinical guidelines for treatment of patients with depression and patients with suicidal behaviours, two years after an actively supported implementation.

**Methods:**

Six psychiatric clinics in Stockholm, Sweden, participated in an implementation of the guidelines. The guidelines were actively implemented at four of them, and the other two only received the guidelines and served as controls. The implementation activities included local implementation teams, seminars, regular feedback, and academic outreach visits. Compliance to guidelines was measured using quality indicators derived from the guidelines. At baseline, measurements of quality indicators, part of the guidelines, were abstracted from medical records in order to analyze the gap between clinical guidelines and current practice. On the basis of this, a series of seminars was conducted to introduce the guidelines according to local needs. Local multidisciplinary teams were established to monitor the process. Data collection took place after 6, 12, and 24 months and a total of 2,165 patient records were included in the study.

**Results:**

The documentation of the quality indicators improved from baseline in the four clinics with an active implementation, whereas there were no changes, or a decline, in the two control clinics. The increase was recorded at six months, and persisted over 12 and 24 months.

**Conclusions:**

Compliance to the guidelines increased after active implementation and was sustained over the two-year follow-up. These results indicate that active local implementation of clinical guidelines involving clinicians can change behaviour and maintain compliance.

## Background

Transferring research results into routine clinical practice is complicated; several studies have described implementation difficulties and the complexity of achieving performance change in health care [1,2]. Single interventions are not effective solutions [3,4]. Although knowledge about effective implementation strategies has increased their use, it has mostly only resulted in small to moderate improvements. Clinical practice guidelines are defined as 'systematically developed statements to assist practitioner and patient decisions about appropriate healthcare for specific clinical circumstances' [5]. Clinical guidelines can be used as tools [6-8], but a passive dissemination alone has rarely been effective in changing health care professionals' behaviour [1,9]. Guidelines have modest influence on clinical practice unless they are successfully integrated into the clinical settings [10]. Guidelines aim to influence the treatment behaviour of practitioners. However, studies are needed to show that physicians exposed to guidelines provide better treatment [11].

There is a gap between evidence-based knowledge and current practice in many medical areas [9,12], and how best to implement guidelines into routine care remains unclear [13]. Implementation of guidelines mostly entails complex interventions, and effective interventions are often elaborated in complicated procedures [14,15]. Commonly evaluated multifaceted implementation strategies are audits and feedback, reminders, and educational outreach [2].

Successful implementation is not enough; there is also a need for continuous follow-up both of compliance to the guidelines and whether it is maintained over time. There are numerous studies showing that compliance returned to baseline after implementation of clinical guidelines [16]. So far, little has been accomplished regarding strategies for maintaining compliance. Objective measures are needed, *e.g*., quality indicators. Ideally, these should be derived from clinical guidelines that are based on scientific research or consensus among experts. These indicators should be measures of process and thus also measure quality of care [17]. Numerous indicators have been developed to evaluate and assess the care provided to patients with chronic physical illnesses [18], but there is lack of studies of care provided to patients with psychiatric disorders [19]. In addition, we have not found long-term follow-up studies in psychiatry on whether changes in practice after guidelines' implementation are sustained.

This study aimed to assess the effects at 12 and 24 months of an implementation intervention designed to improve documentation of quality indicators in accordance with clinical guidelines for treatment of depression and suicidal behaviour in patients at six clinics in Stockholm, Sweden.

## Methods

### Implementation of psychiatric guidelines in Stockholm

In Stockholm county, Sweden, a series of regional clinical guidelines regarding psychiatric disorders has been published and disseminated since 2002 [20,21]. Providers and purchasers in collaboration with Stockholm Medical Advisory Board run the development work. The intention is to require the clinical guidelines to be implemented in all psychiatric clinics in the county in order to provide high quality care on equal terms for all of the county's citizens [20,21]. A pilot study has been conducted on the implementation of clinical guidelines in the care of depression and suicide. Quality indicators derived from the clinical guidelines were used to study compliance. Our previous study showed that the indicators were feasible for audit and feedback as part of the implementation strategy, and a six-month follow-up showed favourable changes in clinical practice [22].

### Settings and participating clinics

In the present study, clinical guidelines for assessment and treatment of depression and guidelines for assessment and treatment of patient with suicidal behaviours were implemented in six psychiatric clinics in Stockholm, Sweden. In Stockholm, treatment is provided almost exclusively by clinics in the public sector. All six psychiatric departments in Stockholm County were invited to implement the guidelines, and four departments decided to participate. Six general psychiatric clinics for adults were included; all were outpatient clinics in an urban area. The resources and organization were comparable. The two departments that declined participation did not differ from the ones that accepted participation in terms of organization of care, personnel resources, and population, as they had uniform contracts with the county council purchasing office. Six clinics in the four departments were randomly selected, and they were randomly assigned to an intervention group or a control group. Two of these clinics participated in implementing the clinical guidelines for depression, and two clinics in implementing the clinical guidelines for suicidal behaviours. Two clinics received the guidelines, but were not included in the intervention and acted as controls.

### Implementation process at the intervention clinics

The study began in May 2003. The first author and an external psychiatrist supported the implementation process during the first six months. Local multidisciplinary teams, co-led by the external psychiatrist, including nurses, physicians, counsellors, and psychologists were established at each of the four active clinics. The teams were locally elected and participation in the local implementation work and meetings was voluntary. The first author presented the implementation study and the quality indicators for each team.

Implementation started with a baseline collection of quality indicators from medical records in order to analyze the gap between clinical guidelines and current local practice. On the basis of this, a series of seminars was conducted to introduce the guidelines according to the identified needs. The implementation teams learned to use strategies for improvements, *e.g*., following a cyclical process of change (plan-do-study-act model) approach [23], which was used to change local practices. Regular meetings then took place and the leaders of the teams promoted the value of implementation activities regarding patient sessions and clinical behaviour. At the meetings, all members of staff were involved in setting local goals for implementation based on the quality indicators. They were also encouraged to provide feedback and identify potential barriers and promoters to change. Feedback was given every month, based on the indicator scores, in order to ensure that improvements were gradually achieved and maintained. Local workshops at the clinics were conducted weekly during the study period, in which participants met to exchange useful approaches.

**Figure 1:**
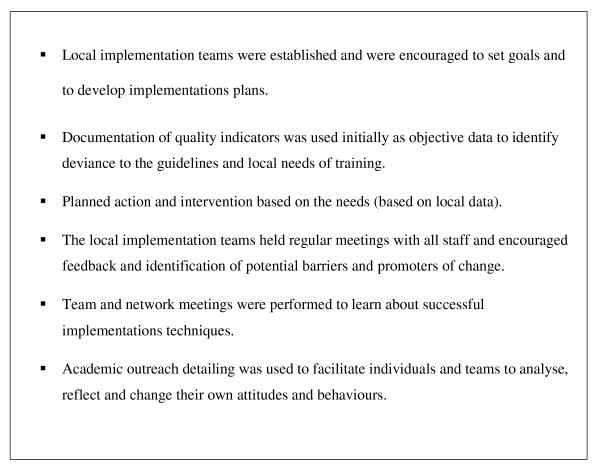
**A summary of the performed implementation interventions**.

The active implementation strategies were based on organizational learning theory and previous knowledge of effective measures to change clinical practice. A learning organisation is described as a process of increasing the capacity for effective organisational action through knowledge and understanding [24]. Through the learning of individuals, the organisational routines are changed. One member of the research team (first author) performed site visits (academic outreach detailing) every month to the intervention clinics during the implementation period. Regular discussions of 'best practice' were held. Through facilitation, practitioners were helped to formulate and reflect on their practical knowledge and professional behaviour. Members of the local implementation teams participated twice in a regional network in order to enhance effective implementation strategies and experience during the study. The participants were encouraged to contact others in the network to exchange experiences and inspiration in the implementation work. During implementation, the adaptation of care defined by clinical guidelines was conducted by the implementation teams. A protocol for local use was developed to promote the adaptation of best practice, based on the clinical guidelines. A summary of the performed interventions is presented in Figure 1.

### Data collection

The data collection took place before the start of the study, and after 6, 12, and 24 months. Patient records from adult men and women with an ICD-10 or DSM-IV diagnosis of depression were eligible for inclusion in the study on the implementation of the clinical guidelines for depression. For the implementation of the clinical guidelines for suicide attempters, the inclusion criteria were patient records from adult men and women appraised at psychiatric emergency clinics after a suicide attempt. The first 120 medical records that fulfilled the inclusion criteria from specific dates were randomly selected from each clinic, identified through the administration system. This was repeated at 6, 12, and 24 months. For the data collection before implementation, 60 to 61 records were collected from each clinic. At the control clinics, 120 medical records were selected before implementation and 120 records at each data collection point during the follow-up period. Trained abstractors examined the medical records. Inter-rater reliability was assessed by a random replicate sample of 40 records. (Kappa 0.92 to 1.0). The study was approved by The Central Ethical Review Board at Karolinska Institutet.

### Study population

A total of 2,165 patient records were included in the systematic assessment. The study of the implementation of the clinical guidelines for depression included 1,083 adult patients, mean age 36.3 years (SD 11.2) diagnosed with a depression according to ICD-10 or DSM-IV [25,26]. There were no differences between the implementation and control clinics regarding gender and age distribution of the included patients. The study of the implementation of the clinical guidelines for suicidal behaviours included 1,082 adult patients, mean age 35.1 years (SD 14.7) At baseline, the mean age of the patients at the implementation clinics was lower (32.5 (12.2), versus 38.3(15.2), t = 2.8, p < 0.01) but there were no gender differences. At six months, there were no age or gender differences.

At 12 and 24 months there were more females and younger patients at the implementation clinics (74.6% versus 64.2%, Chi-square = 4.2, p < 0.1) (mean age 33.7(13.2) versus 40.4(19.0), t = 3.9, p < 0.001), (70.0% versus 52.5%, Chi-square = 10.7, p < 0.001)(mean age 33.3(13.4) versus 37.8(16.7), t = 2.7, p < 0.01).

### Selection of quality indicators

Process indicators extracted from the clinical guidelines were used as indicators of compliance. A modified audit instrument by Gardulf and Nordström [27] was used to assess the presence of the quality indicators. Each indicator was rated on a assessment scale from zero to two. The presence of the quality indicators in the medical records was given a score from zero to two, (zero, recommended criteria to guidelines were not met; one, recommended criteria were partially met according to the definition; and two, a clear occurrence). In a subsequent analyses, we used the quality indicators a binary variables where one and two (*i.e*., partial or full adjustment to the recommendation) were compared to zero (*i.e*., no adjustment to the recommendation). As a sensitivity analysis, quality indicators were also categorised as two versus zero and one. We have found essentially similar results with this alternative approach (data not shown). For all indicators, higher scores were desirable and indicated a better compliance to the guidelines. The indicators also were summarised to a total score for each clinical guideline. The total score for the guidelines for treatment of depression was 22 points and 26 points for the guidelines for suicidal behaviour. Special recording forms were developed for the data collection. Quality indicators for implementation of the clinical guidelines for the care of persons affected by depression and clinical guidelines for suicidal patients are listed in Table 1.

**Table 1:** Quality indicators for evaluation of quality of care in depression treatment and care after a suicide attempt.

Indicator	Definition	Requirements
Accessibility/wait time	The time between referral and actual contact with mental health service	Patients receive an assessment from a mental health specialist within three weeks of their first visit to the outpatient clinic. Patients with depression and suicidal thoughts offered first contact (appointment) within 24 hours.
Diagnostic assessment	Documentation of present depression symptoms. The medical record should document at least three of nine DSM-IV target symptoms for major depression.	Depression symptoms (such as decreased socialization, sleep disorders, poor appetite according DSM-IV) noted in the medical record.
Standardized rating scale	Clinical depression assessment that includes a standardized rating scale.	Monitoring signs and symptoms of depression using a validated standardized rating scale at the first visit. Scale and total sum documented in the medical record. Suggestions of scales to be used were presented in the guidelines.
Diagnostic instrument	Diagnostic structured interview	A semi-structured diagnostic interview *e.g*., SCID or M.I.N.I performed. Completed before the third visit.
Standardized rating scale during treatment	Standardized rating scale during treatment for assessment of symptoms and behaviour.	Standardized rating scale performed within two weeks. Monitoring signs and symptoms of depression using standardized rating scale during treatment. Adjusted interventions if signs and symptoms are still present, presented in the guidelines.
Substance, drug abuse	Screening for substance use disorder.	Asked for current substance use and evaluated for the presence and/or history of substance use disorder. Screenings instruments such as AUDIT. Motivation interview conducted *e.g*., CAGE method.
Treatment plan (care plan)	A written treatment plan documented and individually tailored for the patient	The treatment plan should include; treatment, goals, time for evaluation and drawn up together with the patient.
Evaluation/Outcome	Has patient responded to antidepressant? Achieved symptom remission or reduction between admission and follow-up?	Documented response to treatment within expected treatment frame and monitored progress. Completed a comprehensive evaluation of symptoms.
Continuity	Ability to provide uninterrupted care over time.	Continuity offered to the patient, same caregiver during treatment. Defined as less than two different caregivers.
Suicide assessment	A structured assessment documented in the medical record using standardized rating scale.	Identified suicidal thoughts, plans and symptoms, documented and evaluated in the medical record. Re-screen and assessment performed at every visit and documented in the medical record.
Antidepressant medication	Current treatment with an antidepressant medication for patients with major depressive disorder, moderate or severe.	Begin appropriate antidepressant medication according the guidelines. Started within two visits.
Specialist assessment after suicide attempt	Assessment by a senior physician within 24 hours after a suicide attempt	A senior mental health specialist has made the assessment within 24 hours.
Suicide assessment	A structured assessment documented in the medical record using standardized rating scales.	Identified suicidal thoughts, plans and symptoms, documented and evaluated in the medical record. Depression assessment conducted using standardized rating scale.
Follow-up	Care plan formulated and documented.	Documented discharge plans. Referral to a psychiatric outpatient clinic
Evaluation	Documented assessment after discharge.	Should have a follow-up visit with a mental health specialist within one week after assessment or discharge. Telephone contact with patient during this period.

### Statistical analysis

The data were analysed using STATA and SPSS for Windows, versions 10 and 16.0, respectively. Inter-rater reliability was analysed by calculating Cohen's Kappa. Differences regarding age and gender distribution of the included patient records at implementation and control clinics were analysed using chi-square test and T-tests. To address the nested structure of our data, we fitted random-effects logit models where we clustered patients within their health care providers using 'xtlogit' command in STATA [28]. Odds ratios were calculated for the dichotomized quality indicators comparing quality of care before (reference category) and after 6, 12, and 24 months, respectively.

## Results

### Compliance to the clinical guidelines for depression

Table 2 shows compliance at baseline, and 6, 12, and 24 months after implementation of clinical guidelines for depression, based on the quality indicators. The documentation of the quality indicators improved from the baseline in the four clinics where implementation was carried out, whereas there were no changes, or a decline, in the documentation of most quality indicators in those without implementation. For most of the quality indicators, the increase was recorded at six months and persisted over 12 and 24 months. Although, for a few quality indicators the 24-month follow-up audit showed a slight decrease compare to the measurement at 12 months.

**Table 2:** The compliance before, 6, 12, and 24 months after the implementation of clinical guidelines for depression in % (n).

	Implementation clinics		Control clinics	
**Indicator**	**% (n)**	**OR (95% CI)**	**% (n)**	**OR (95% CI)**

Accessibility/wait time				
0 months	77.9 (95)	reference	59.0 (36)	reference
6 months	89.2 (107)	2.4 (1.1-5.2)	53.3 (32)	0.6 (0.3-1.4)
12 months	97.1 (233)	13.4 (5.3-34.0)	44.2 (53)	0.4 (0.2-0.9)
24 months	90.0 (216)	2.5 (1.3-4.9)	51.7 (62)	0.6 (0.3-1.2)
Diagnostic assessment				
0 months	83.6 (102)	reference	88.5 (54)	reference
6 months	97.5 (117)	9.6 (2.5-36.1)	90.0 (54)	1.1 (0.4-3.6)
12 months	97.5 (234)	11.1 (4.0-30.9)	83.3 (100)	0.6 (0.3-1.6)
24 months	97.9 (235)	10.9 (3.7-32.4)	79.2 (95)	0.5 (0.2-1.2)
Diagnostic instrument				
0 months	12.3 (15)	reference	1.6 (1)	reference
6 months	28.3 (34)	2.8 (1.4-5.5)	0	na
12 months	41.3 (99)	5.3 (2.9-9.7)	0.8 (1)	na
24 months	44.2 (106)	5.7 (3.1-10.5)	0.8 (1)	na
Standardized rating scale				
0 months	64.8 (79)	reference	44.3 (27)	reference
6 months	91.7 (110)	6.2 (2.9-13.3)	33.3 (20)	0.7 (0.3-1.4)
12 months	95.0 (228)	11.1 (5.5-22.3)	37.5 (45)	0.8 (0.4-1.5)
24 months	94.2 (226)	9.1 (4.7-17.6)	36.7 (44)	0.7 (0.4-1.4)
Standardized rating scale during treatment				
0 months	50.0 (61)	reference	24.6 (15)	reference
6 months	87.5 (105)	7.6 (3.9-14.9)	38.3 (23)	1.9 (0.8-4.2)
12 months	97.5 (234)	47.5 (19.0-118.2)	30.8 (37)	1.4 (0.7-2.8)
24 months	88.3 (212)	8.1 (4.7-14.3)	33.3 (40)	1.5 (0.8-3.1)
Substance/drug abuse				
0 months	46.7 (57)	reference	32.8 (20)	reference
6 months	87.5 (105)	8.0 (4.2-15.4)	53.2 (32)	2.8 (1.3-6.2)
12 months	94.2 (226)	18.5 (9.7-35.4)	35.0 (42)	1.2 (0.6-2.3)
24 months	88.8 (213)	9.1 (5.3-15.6)	43.3 (52)	1.8 (0.9-3.6)
Treatment (care) plan				
0 months	59.8 (73)	reference	42.6 (26)	reference
6 months	87.5 (105)	5.5 (2.7-11.1)	38.3 (23)	0.9(0.4-1.9)
12 months	90.4 (217)	8.4 (4.5-15.5)	34.2 (41)	0.7 (0.4-1.4)
24 months	91.3 (219)	8.1 (4.3-15.0)	27.5 (33)	0.5 (0.3-1.0)
Evaluation/outcome				
0 months	66.4 (81)	reference	59.0 (36)	reference
6 months	95.8 (115)	11.9 (4.5-31.7)	55.0 (33)	0.8 (0.4-1.7)
12 months	97.5 (234)	20.3 (8.2-49.9)	48.3 (58)	0.6 (0.3-1.1)
24 months	95.8 (230)	11.9 (5.7-25.0)	48.3 (58)	0.6 (0.3-1.1)
Continuity				
0 months	77.0 (94)	reference	78.7 (48)	reference
6 months	95.0 (114)	5.6 (2.2-14.1)	61.7 (37)	0.4 (0.2-1.1)
12 months	99.6 (239)	72.0 (9.7-537.4)	71.7 (86)	0.7 (0.3-1.5)
24 months	95.8 (230)	6.7 (3.1-14.4)	68.3 (82)	0.6 (0.3-1.4)
Suicide assessment				
0 months	40.2 (49)	reference	45.9 (28)	reference
6 months	95.8 (115)	36.1 (13.5-96.5)	35.0 (21)	0.6 (0.3-1.4)
12 months	93.8 (225)	23.3 (12.1-44.7)	35.8 (43)	0.7 (0.4-1.2)
24 months	97.5 (234)	61.3 (24.8-151.9)	30.0 (36)	0.5 (0.3-1.0)
Antidepressant medication				
0 months	54.1 (66)	reference	45.9 (28)	reference
6 months	90.8 (109)	8.3 (4.1-17.0)	36.7 (22)	0.7 (0.3-1.4)
12 months	85.4 (205)	5.0 (3.0-8.2)	44.2 (53)	1.0 (0.5-1.7)
24 months	92.5 (222)	10.3 (5.7-18.8)	41.7 (50)	0.8 (0.4-1.5)

^na ^The numbers did not allow calculations.

Odds ratios adjusted for age and gender with baseline as the reference is presented with CI (95%)

**Figure 2:**
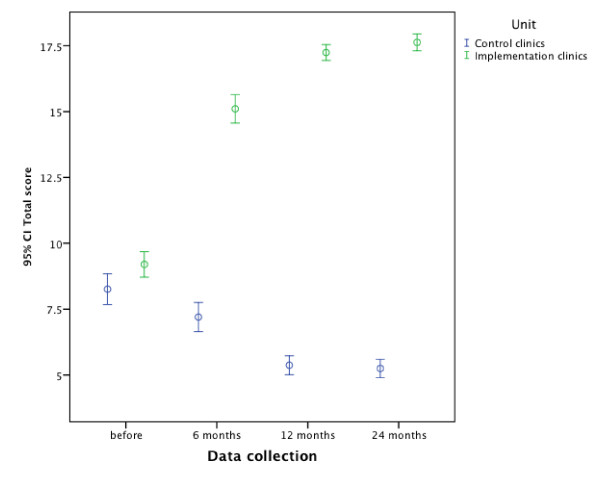
**Total score of quality indicators for clinical guidelines for depression and suicide**.

The compliance for some indicators was low initially and after implementation showed considerable improvement, *e.g*., the compliance for structured suicide assessment rose from 40.2% (for a clear occurrence to guidelines) before implementation to at least 97.5% after (Table 2). Total score of the quality indicators for clinical guidelines for depression with 95% confidence interval are presented in Figure 2.

### Compliance to the clinical guidelines for the management of suicide attempters

A similar pattern was seen in the documentation of the quality indicators in the clinics that implemented the clinical guidelines for suicide attempters. There was an increase of the documentation at six months, and the increase persisted over 12 and 24 months (Table 3).

**Table 3:** The compliance before, 6, 12, and 24 months after the implementation of clinical guidelines for suicidal behaviour in % (n).

	Implementation clinics		Control clinics	
**Indicator**	**% (n)**	**OR (95% CI)**	**% (n)**	**OR (95% CI)**

Accessibility/wait time				
0 months	15.7 (19)	reference	29.5 (18)	reference
6 months	14.2 (17)	0.9 (0.4-1.8)	31.7 (19)	1.1 (0.5-2.4)
12 months	70.4 (169)	13.7 (7.7-24.4)	0	na
24 months	59.2 (142)	8.3 (4.7-14.6)	0	na
Diagnostic assessment				
0 months	49.6 (60)	reference	26.2 (16)	reference
6 months	73.3 (88)	2.9 (1.7-5.0)	16.7 (10)	0.6 (0.2-1.5)
12 months	83.3 (200)	5.4 (3.2-8.9)	0.8 (1)	0 (0.0-0.2)
24 months	91.7 (220)	11.8 (6.5-21.2)	0	na
Diagnostic instrument				
0 months	0	reference	0	reference
6 months	7.5 (9)	na	0	na
12 months	0	na	0	na
24 months	7.5 (18)	na	0	na
Standardized rating scale				
0 months	41.3 (50)	reference	27.9 (17)	reference
6 months	67.5 (81)	3.0 (1.7-5.0)	16.7 (10)	0.5 (0.2-1.4)
12 months	79.2 (190)	5.5 (3.4-8.9)	0	na
24 months	78.3 (188)	5.2 (3.2-8.4)	0.8 (1)	0.0 (0.0-0.2)
Standardized rating scale during treatment				
0 months	16.5 (20)	reference	16.4 (10)	reference
6 months	52.5 (63)	5.8 (3.2-10.5)	10.0 (6)	0.6 (0.2-1.7)
12 months	22.9 (55)	1.6 (0.9-2.7)	0.8 (1)	0.04 (0.02-0.35)
24 months	55.8 (134)	6.6 (3.8-11.3)	5.0 (6)	0.3 (0.1-0.9)
Substance/drug abuse				
0 months	52.1 (63)	reference	55.7 (34)	reference
6 months	64.2 (77)	1.7 (1.0-2.9)	56.7 (34)	1.0 (0.5-2.1)
12 months	77.5 (186)	3.4 (2.1-5.5)	25.0 (30)	0.3 (0.2-0.5)
24 months	80.0 (192)	3.8 (2.4-6.2)	29.2 (35)	0.3 (0.1-0.6)
Treatment (care) plan				
0 months	37.4 (68)	reference	44.3 (27)	reference
6 months	58.9 (106)	4.4 (2.5-7.6)	41.7 (25)	0.9 (0.4-1.9)
12 months	67.1 (161)	4.4 (2.7-7.1)	0.8 (1)	0.0 (0.0-0.1)
24 months	79.2 (190)	8.0 (4.9-13.3)	0.8 (1)	0.0 (0.0-0.1)
Evaluation/outcome				
0 months	20.7 (25)	Reference	19.7 (12)	reference
6 months	47.5 (57)	3.5 (2.0-6.2)	8.3 (5)	0.4 (0.1-1.2)
12 months	25.8 (62)	1.3 (0.8-2.3)	0	na
24 months	51.7 (124)	4.1 (2.5-6.9)	0	na
Continuity				
0 months	86.0 (104)	reference	49.2 (30)	reference
6 months	81.7 (98)	0.7 (0.4-1.5)	31.7 (19)	0.5 (0.2-1.1)
12 months	96.3 (231)	4.2 (1.8-9.9)	0	na
24 months	91.3 (219)	1.7 (0.9-3.5)	0	na
Suicide assessment				
0 months	55.4 (67)	reference	82.0 (50)	reference
6 months	93.3 (112)	13.6 (5.9-31.5)	73.3 (44)	0.6 (0.2-1.4)
12 months	87.1 (209)	6.1 (3.5-10.6)	50.0 (60)	0.2 (0.1-0.5)
24 months	97.1 (233)	33.6 (14.1-80.2)	56.7 (68)	0.3 (0.1-0.6)
Specialist assessment				
0 months	50.4 (61)	reference	83.6 (51)	reference
6 months	85.4 (103)	6.5 (3.4-12.3)	83.3 (50)	1.0 (0.4-2.6)
12 months	87.5 (210)	7.5 (4.4-12.9)	86.7 104)	1.3 (0.5-3.0)
24 months	91.7 (220)	11.8 (6.5-21.5)	71.7 (86)	0.5 (0.2-1.1)
Follow-up				
0 months	72.7 (88)	reference	75.4 (46)	reference
6 months	88.3 (106)	2.9 (1.5-5.8)	65.0 (39)	0.6 (0.3-1.4)
12 months	86.3 (207)	2.4 (1.4-4.1)	34.2 (41)	0.2 (0.1-0.3)
24 months	92.1 (221)	4.5 (2.4-8.3)	37.5 (45)	0.2 (0.1-0.4)
Evaluation assessment				
0 months	32.2 (39)	reference	18.0 (11)	reference
6 months	64.2 (77)	4.0 (2.3-6.9)	13.3 (8)	0.6 (0.2-1.6)
12 months	63.8 (153)	3.8 (2.4-6.1)	6.7 (8)	0.3 (0.1-0.8)
24 months	75.0 (180)	6.8 (4.2-11.2)	10.8 (13)	0.4 (0.2-1.1)

^Odds ratios adjusted for age and gender with baseline as the reference is presented with CI (95%)^

^na ^The numbers did not allow calculations.

Some indicators were more sensitive to change, *e.g*., structured suicide assessment for suicidal patients rose from 55.4% to 97.1% for a partial or clear occurrence of guidelines and specialist assessment rose from 50.4% to 91.7%. Figure 2 shows the total score of the quality indicators for clinical guidelines for suicidal behaviours with a 95% confidence interval.

## Discussion

This paper describes an actively supported implementation of clinical guidelines in psychiatric settings and examined compliance before implementation and after 6, 12, and 24 months using quality indicators as measurements. The results showed that there was a consistent significant increase in the documentation of almost all of the quality indicators, that this occurred after a rather short period of time, and was sustained at almost the same level throughout the two-year study period. The increase was only observed in the intervention clinics and not at the clinics to which the guidelines were only disseminated. These findings imply that a systematic implementation approach gives sustainable change, at least over a two-year period, as documented by quality indicators. Our study describes the challenge implicit in real-world implementation aimed at improving the quality of care. The aim of all implementation is a change that remains after the support is withdrawn, and the results indicate that changes had taken place in the organization and structure of the care provided at the implementation clinics. In order to achieve these changes, an active implementation was needed and not just a dissemination of, or lecturing about, guidelines. This finding is in accord with earlier studies [11,29,30].

It could be assumed that the current clinical practice was close to recommended care as presented in the guidelines, because the latter were based on information easily available to all clinicians. However, we found that there were large gaps between current clinical practice and recommended practice according to guidelines, especially in the clinics where guidelines for suicidal patients were implemented. The implementation required complex changes in clinical practice, better collaboration, and changes in the organization of care.

There are several likely explanations for the observed improvements. First, local implementation teams with multidisciplinary members were established. This initiative was intended to develop collaboration for organizational learning of best practice and change of clinical practice. The teams were encouraged to involve all staff at the clinic in adapting the guidelines for local use. Using local teams facilitated collaborative partnerships, integrated knowledge, and action. Thus, the team members gained a deeper understanding of the context and challenges of the local health service.

Further, the interventions included audits and regular feedback, which helped the local teams to monitor the implementation. The aim was that the local teams would be able to choose the most important areas for intervention and to measure success in terms of improved compliance to the guidelines and outcomes. Previous studies have reported that this enhances learning and facilitate translation of insight to daily work [31,32]. The organization should make use of the change process to implement changes of proven effectiveness regarding implementations strategies.

The feedback was based on quality indicators that were easy to use and showed a high inter-reliability. The indicators were all process indicators that had previously been the subject of discussion as to how to use them more effectively in mental health care, and they were not particularly controversial [33]. Furthermore, the changes are unlikely to be sustained if implementation does not include repeated measurements to access advancement and encourage modifications.

Another active strategy was that an outside researcher made regular visits to support the local teams. Moreover, all involved teams were part of a regional network that held regular meetings, because successful adoption of innovations often depends on interpersonal relationships within a system or an organization. An organization that supports knowledge sharing, and encourages observation and reflections is more successful at innovation and diffusion [34]. The network, as well as the visits, facilitated this. Although the teams worked locally, they were able to learn about organizational culture, implementation technique, and improvement models from colleagues in the regional network. Moreover, this supported the involved practitioners in analyzing, reflecting upon, and changing their own attitudes and behaviours. The goal was to transfer implementation technology into the participating organizations in order to continuously improve each organization's capacity for change.

Another critical issue for success of a diffusion of innovation strategy is leadership [35]. Leadership is described as an important factor in translating guidelines into clinical practice. Lack of support from leadership is identified as one of the greatest barriers [36]. According to Garside [37], leaders must continually show the desired direction of change, and support the staff in their new roles and new skills in a change of organisation or process. In the present study, the leadership was involved at an initial meeting at which the guidelines were presented. Because they had all volunteered to participate, they supported the implementation activities and created a culture in which the changes in clinical practice were possible.

Thus, a multifaceted intervention including a variety of active strategies was used [3], which previously has been reported to be more effective than passive strategies or just the use of feed-back or audit [38]. Shortell *et al*. [39] have suggested that five dimensions are needed for a successful implementation, *i.e*., process, strategic, cultural, technical, and structural. Our implementation program included all of these dimensions.

The standard of care is not the same as the quality of care. The quality of care provided by the clinician may be below, equal to, or even above the acceptable standard of care. Practice parameters are strategies for patient management, designed to assist health care professionals in clinical decision-making. The practice parameters describe the generally accepted practices, but are not intended to define a standard of care. The intentions with the quality indicators as presented in the clinical guidelines were to represent ideal practice. Thus, they could be used to measure deficiencies between current practice and ideal practice as defined in the guidelines, which would indicate an area for intervention. These practice parameters reflect the state of knowledge at the time of development of the guidelines, and most certainly need to be regularly updated.

Psychiatric disorders are of great importance in public health. Depression is now the fourth-leading cause of the global disease burden and the leading cause of disability worldwide. Depression is the most important risk factor for suicide, which is among the top three causes of death in young people ages 15 to 35 [40]. Depression seriously reduces the quality of life for individuals and their families, and often aggravates the outcome of other physical health problems. Because depression is highly treatable, and currently undertreated, it is an appropriate focus for improvement of the treatment by implementing available evidenced-based clinical guidelines. Guideline implementation studies in the care of psychiatric disorders are lacking, but a review by Weingartner of clinical guidelines in chronic medical diseases has stressed the importance of multifaceted interventions [41]. A comparable conclusion that multiple strategies seem to be most effective is presented in a systematic meta-review by Francke [16].

There were some limitations in the present study. Firstly, although both intervention and control clinics were randomly assigned, all had volunteered to participate, and therefore probably were more motivated to change. Secondly, given the fact that clinical practice change is a complex phenomenon dependent on local context, results from one particular setting can be generalised only with great caution [42].

Our study had a cluster design where patients were nested within their health care providers, and the health care providers were nested within their clinics. While the clustering at the provider level was properly addressed in our analyses, due to the low number of participating clinics it was not possible to fit a three-level model. Therefore, we could not investigate the possible role of clinic level covariates, and the lack of controlling for autocorrelation within clinics might inflate somewhat the standard error of our estimates.

Addressing local needs when implementing clinical guidelines is important in closing the gap between research and practice. The need to adapt implementation efforts to local circumstances has been shown to be valuable [43]. Adequate funding is needed to train the staff in the intervention techniques, establish protocols, and support evaluation of the outcome. Further research is needed on practical frameworks to facilitate the implementation of intervention in mental health care settings.

A large number of factors determine whether or not implementation will be successful and all factors cannot be addressed within one theory or model of change. Further studies are needed to examine our implementation approach with reference to theories about the implementation of change. The strength of the present study is that it is, to our knowledge, the first one to assess the long-term effects of implementation of psychiatric guidelines.

## Conclusions

This study suggested that the compliance to clinical guidelines, for treatment of depression and suicidal behaviour, was implemented and sustained over a two-year period after an active implementation. Quality indicators were helpful tools in the implementation process as well as in the evaluation. Thus, supported local implementation based on local organisation theory may be a strategy for narrowing the gap between evidence-based care and current practice.

## Competing interests

The authors declare that they have no competing interests.

## Authors' contributions

TF, AÅW, MB, and YF have all participated to the design of the study. TF, YF, IJ, and APL have analyzed the data. All authors participated in interpretation of the results. TF drafted the manuscript and all other authors provided critical revision of the draft for important intellectual content. All authors read and approved the final manuscript.
